# The psychological effects of COVID-19 on hospital workers at the beginning of the outbreak with a large disease cluster on the Diamond Princess cruise ship

**DOI:** 10.1371/journal.pone.0245294

**Published:** 2021-01-11

**Authors:** Keiko Ide, Takeshi Asami, Akira Suda, Asuka Yoshimi, Junichi Fujita, Munetaka Nomoto, Tomohide Roppongi, Kousuke Hino, Yuichi Takahashi, Kaori Watanabe, Tomoko Shimada, Toyoko Hamasaki, Emi Endo, Tomoko Kaneko, Michiko Suzuki, Kazumi Kubota, Yusuke Saigusa, Hideaki Kato, Toshinari Odawara, Hideaki Nakajima, Ichiro Takeuchi, Takahisa Goto, Michiko Aihara, Akitoyo Hishimoto

**Affiliations:** 1 Department of Psychiatry, Yokohama City University Hospital, Yokohama, Japan; 2 Department of Psychiatry, Yokohama City University School of Medicine, Yokohama, Japan; 3 Health Management Office, Yokohama City University Hospital, Yokohama, Japan; 4 Department of Child Psychiatry, Yokohama City University Hospital, Yokohama, Japan; 5 Psychiatric Center, Yokohama City University Medical Center, Yokohama, Japan; 6 Advanced Emergency Medical Service Center, Yokohama City University Medical Center, Yokohama, Japan; 7 Yokohama East Area Habilitation Center for Children, Yokohama, Japan; 8 Nursing Department, Yokohama City University Hospital, Yokohama, Japan; 9 Patient Care and Safety Management Department, Yokohama City University Hospital, Yokohama, Japan; 10 Nursing Department, Yokohama City University Medical Center, Yokohama, Japan; 11 Department of Biostatistics, Yokohama City University Graduate School of Medicine, Yokohama, Japan; 12 Infection Prevention and Control Department, Yokohama City University Hospital, Yokohama, Japan; 13 Department of Hematology and Clinical Immunology, Yokohama City University School of Medicine, Yokohama, Japan; 14 Health Management Center, Yokohama City University, Yokohama, Japan; 15 Advanced Critical Care Medicine, Yokohama City University Hospital, Yokohama, Japan; 16 Department of Anesthesiology and Critical Care Medicine, Yokohama City University Graduate School of Medicine, Yokohama, Japan; 17 Department of Environmental Immuno-Dermatology, Yokohama City University Graduate School of Medicine, Yokohama, Japan; Chiba Daigaku, JAPAN

## Abstract

The aim of the present study was to investigate the psychological effects of the COVID-19 outbreak and associated factors on hospital workers at the beginning of the outbreak with a large disease cluster on the Diamond Princess cruise ship. This cross-sectional, survey-based study collected demographic data, mental health measurements, and stress-related questionnaires from workers in 2 hospitals in Yokohama, Japan, from March 23, 2020, to April 6, 2020. The prevalence rates of general psychological distress and event-related distress were assessed using the 12-item General Health Questionnaire (GHQ-12) and the 22-item Impact of Event Scale-Revised (IES-R), respectively. Exploratory factor analysis was conducted on the 26-item stress-related questionnaires. Multivariable logistic regression analysis was performed to identify factors associated with mental health outcomes for workers both at high- and low-risk for infection of COVID-19. A questionnaire was distributed to 4133 hospital workers, and 2697 (65.3%) valid questionnaires were used for analyses. Overall, 536 (20.0%) were high-risk workers, 944 (35.0%) of all hospital workers showed general distress, and 189 (7.0%) demonstrated event-related distress. Multivariable logistic regression analyses revealed that ‘Feeling of being isolated and discriminated’ was associated with both the general and event-related distress for both the high- and low-risk workers. In this survey, not only high-risk workers but also low-risk workers in the hospitals admitting COVID-19 patients reported experiencing psychological distress at the beginning of the outbreak.

## Introduction

On February 3, 2020, an outbreak of novel coronavirus disease (COVID-19) caused by severe acute respiratory syndrome coronavirus 2 (SARS-CoV-2) was reported on Cruise Ship Diamond Princess quarantined in the Yokohama Port in Japan [1]. Every day, the media reported the increasing number of patients who tested positive for SARS-CoV-2 on the cruise ship, labeled as a “second Wuhan,” where about one in five people aboard became infected [2]. Since the media continuously emphasized the effects of COVID-19 infection, the public plunged into anxiety and fear. Hospital workers in Yokohama likely experienced huge psychological stress, despite the fact that the COVID-19 infection had not exploded in Japan yet. Wards for COVID-19 and their staff had particularly limited capacity in hospitals in Yokohama. No hospital closures or quarantine of staff was in place at that time.

On February 6, one of our two hospitals, Yokohama City University Medical Center (Medical Center), admitted its first COVID-19 patient. On February 9, another hospital, Yokohama City University Hospital (University Hospital), admitted its first COVID-19 patient. The University Hospital and the Medical Center accepted 11 and 9 patients, respectively, from the Diamond Princess since then [3, 4].

We felt that the hospital workers experienced much more severe emotional distress than ever, not only high-risk workers (e.g., working in the ward for COVID-19) but also low-risk workers (e.g., working in the department without COVID-19 patients). We also thought that this distress was related to the amount of exposure to TV news and Internet media reporting about the Diamond Princess and the pandemic in China repeatedly because there were only a few patients with COVID-19 in our hospitals since it was the beginning of the outbreak.

Many previous studies have reported the effects of the outbreak of infections on mental health in hospital workers, such as 2003 SARS [5, 6], 2009 (H1N1) influenza [7, 8], and 2015 MERS [9]. Regarding the COVID-19, mental health outcomes and associated factors among health care workers have already been reported in China [10, 11] and many other countries worldwide [12–14]. The influence of media exposure on mental health during the COVID-19 pandemic has also been reported [15]. Those studies have, however, focused on psychological well-being mostly among health care workers directly exposed to infected patients, and they were conducted after the peak of the outbreak. In addition, most studies conducted during the COVID-19 pandemic used an online survey format, which might reduce the validity of the assessed data. To the best of our knowledge, only a few studies have investigated hospital workers' mental health status at the beginning of the pandemic or mentioned psychological symptoms experienced by both high- and low-risk workers [16, 17].

To clarify the psychological effects of the COVID-19 pandemic on all our hospital workers, we administered paper-based questionnaires to both high- and low-risk workers, including medical doctors, nurses, and individuals in many other positions in our two hospitals.

We speculated that the low-risk workers might be psychologically affected differently compared to the high-risk workers. Therefore, general psychological distress, event-related distress, and their risk factors, which were assessed by the questionnaires, were evaluated separately for high and low-risk workers. We then tried to clarify the psychological effects of the COVID-19 pandemic on hospital workers in each risk environment, and the effects of their work risks, sociodemographic, occupation, and the stress-related factors on their psychological well-being.

The first wave of COVID-19 infection has not peaked in Japan at the time of our research, and only the Diamond Princess cruise ship represented the big disease cluster. Therefore, this survey study was conducted at the beginning of the outbreak, before the peak of the first wave of the COVID-19 pandemic, which is different from other previous studies.

## Methods

### Subjects

We conducted a survey study with all our hospital workers in both the University Hospital and the Medical Center, both teaching hospitals and flagship hospitals in Yokohama. The characteristics of these hospitals are shown in Table 1.

**Table 1:** Hospital characteristics.

	University Hospital	Medical Center
Total no. hospital workers	2115	2018
Medical Doctor	587	436
Nurse	769	944
Other medical professionals	217	303
Office workers and clinical clerks	461	312
Others	81	23
Valid Response No. (%)	1457 (68.9%)	1240 (61.4%)
No. beds	674	726
No. inpatients with COVID-19		
March 23, 2020	6	2
April 6, 2020	3	3
Total no. inpatients with COVID-19		
till March 23, 2020	9	5
till April 6, 2020	14	6
Patients from the Diamond Princess	11	9

Jobs classified as medical doctor; nurse; other medical professionals (clinical laboratory technicians, radiological technologists, medical engineers, pharmacists, dieticians, social workers, physical therapists, occupational therapists, and speech therapists); office workers and clinical clerks; or others (nursing assistants, janitors, food service, and laundry staff).

A questionnaire was distributed to all 4133 hospital workers, and 2915 completed questionnaires (70.5%) were collected. Of these, 218 were excluded because of a lack of informed consent or missing at least one answer on sociodemographic characteristics or psychological rating scales. Therefore, 2697 questionnaires (65.3%) were used for our analyses.

### Procedure

The paper-based, self-administered anonymous questionnaires were personally handed to all hospital workers or placed on their desks or in their mailboxes starting on March 23, 2020. Then, they were collected by the in-house mail system until April 6.

### Content of the questionnaire

The first question asked for informed consent to use the responses in the survey. The questionnaire consisted of four parts, questions assessing sociodemographic characteristics, the 12-item General Health Questionnaire (GHQ-12) [18, 19], the Impact of Event Scale-Revised (IES-R) [20, 21], and stress-related questions associated with COVID-19. Participants were asked whether they worked in the department, such as wards for COVID-19 or ER, where they come into direct contact with COVID-19 patients. Those who responded ‘Yes’ were defined as high-risk workers, and those who answered ‘No’ were defined as low-risk workers.

We calculated total scores for the GHQ-12 using the GHQ scoring method [18] and subsequently divided our hospital workers into two groups, workers ‘with’ or ‘without’ general distress. A threshold of 3/4 was used because the mean score of the GHQ-12 was 3.08 [22]. This threshold is often used in studies with Japanese, while 2/3 is employed in studies conducted in other countries [23, 24].

The IES-R is a self-report, 22-item, 5-point scale, originally developed to assess posttraumatic stress syndrome, and it is used widely to assess the psychological response to a stressful event. We used the IES-R here to assess the ‘event-related distress of the COVID-19 outbreak on the Diamond Princess’. A threshold of 24/25 was used to divide our hospital workers into two groups also, workers ‘with’ or ‘without’ event-related distress [9, 25, 26].

The stress-related questions consisted of 26 items measured on a 4-point Likert scale (0 = never; 1 = rarely; 2 = sometimes; 3 = always) to describe how often they experienced various stressors during that period. Nineteen of the 26 items were adapted from studies on the SARS [24] and the H1N1 influenza pandemics [7]. We added seven original items inquiring about family support (Q11) and increased exposure to TV (Q22) and internet media (Q23). The Cronbach’s alpha coefficient for the 26 stress-related questions was α = 0.87, indicating good internal consistency and acceptable reliability. The Yokohama City University Ethical Review Board (B200200053, B200200054) approved this study, and participation was voluntary.

### Statistical analysis

Descriptive analyses were conducted to examine the characteristics of the participants. Chi-square tests were then carried out to identify factors associated with high GHQ-12 scores and high IES-R scores.

A factor analysis was conducted on the 26 stress-related questions using the maximum likelihood method and Promax rotation because the previous studies have not yet determined the factor structure. The number of factors was determined by the size of the eigenvalue (greater than 1.00) and the relative size of the values according to different factor models. For each of the factors, the total scores of the stress-related questions were calculated.

To determine potential risk factors for general and event-related distress for high- and low-risk workers separately, multivariable logistic regression analyses were conducted in each risk group (forced entry method). ‘Workers with/without general distress, as evaluated by the GHQ-12,’ or ‘Workers with/without event-related distress, as assessed by the IES-R,’ were used as dependent variables, and the demographic data and the total scores of the factors derived from the factor analysis were treated as independent variables. The association between risk factors and outcomes are presented as adjusted odds ratios (AORs) and 95% CIs with the risk factors which are gender, age group, occupation, direct exposure to COVID-19 patients, preexisting disease, living with partner, living with elderly, confident in standard precaution, and the factors1-6. To assess associations between risk factors and event-related distress specific to the COVID-19 outbreak on the Diamond Princess, the scores of the GHQ-12 were included as additional independent variables in the event-related distress models. Data analyses were performed using the SPSS statistical software version 21.0 (IBM Corp) with a significance level set at p < .05 (two-tailed).

## Results

### Descriptive analyses

The first column of Table 2 shows the characteristics of the study participants. In the 2697 hospital workers, 536 (20.0%) were workers at a high-risk for infection of COVID-19. Over one-third of them showed general distress (high score on GHQ-12, n = 944 (35.0%)), even though only 7.0% of them (n = 189) demonstrated event-related distress (high score of IES-R).

**Table 2:** Participants characteristics and associations with general distress and event-related distress.

	Total	General Distress ^a^			Event-related Distress ^b^		
	n = 2697	n = 944 (35.0%)			n = 189 (7.0%)		
Characteristics	No.	No.	(%)	P ^c^	No.	(%)	P ^c^
Hospital							
University	1457	496	34.0		100	6.9	
Medical Center	1240	448	36.1	.274	89	7.2	.763
Work environment							
High-risk worker	536	209	39.0		52	9.7	
Low-risk worker	2161	735	34.0	.034*	137	6.3	.008*
Gender							
male	702	174	24.8		42	6.0	
female	1995	770	38.6	< .001**	147	7.4	.230
Age group (years)							
20–29	625	162	25.9		24	3.8	
30–39	707	250	35.4		39	5.5	
40–49	750	305	40.7		74	9.9	
50-	615	227	36.9	< .001**	52	8.5	< .001**
Occupation							
Medical Doctor	555	146	26.3		17	3.1	
Nurse	1045	399	38.2		75	7.2	
Other medical professionals ^d^	359	113	31.5		15	4.2	
Office workers and clinical clerks	527	216	41.0		55	10.4	
Others^e^	211	70	33.2	< .001**	27	12.8	< .001**
Direct contact with COVID-19 patient							
yes	328	136	41.5		32	9.8	
no or don't know	2369	808	34.1	.01*	157	6.6	.049*
preexisting disease							
yes	191	82	42.9		20	10.5	
no	2506	862	34.4	.018*	169	6.7	.056
Living with partner							
yes	1422	496	34.9		102	7.2	
no	1275	448	35.1	.903	87	6.8	.763
Living with children							
yes	1095	402	36.7		83	7.6	
no	1602	542	33.8	.128	106	6.6	.357
Living with elderly							
yes	363	153	42.1		38	10.5	
no	2334	791	33.9	.003*	151	6.5	.008*
Confident in standard precaution							
yes	1544	494	32.0		94	6.1	
no	1153	450	39.0	< .001**	95	8.2	.033*
Event-related distress							
yes	189	154	81.5		-	-	
no	2508	790	31.5	< .001**	-	-	-

Abbreviations: GHQ-12, the 12-item General Health Questionnaire; IES-R, the impact of Event Scale-Revised.

^a^ General distress evaluated by the GHQ-12 (≥4).

^b^ Event-related distress evaluated by the IES-R (≥25).

^c^ P values were calculated from 2-sided chi-square tests.

^d^ Other medical professionals include clinical laboratory technicians, radiological technologists, medical engineers, pharmacists, dieticians, social workers, physical therapists, occupational therapists, and speech therapists.

^e^ Others include nursing assistants, janitors, food service, and laundry staff.

Two-tailed chi-square tests were performed to evaluate the differences in proportions (Table 2). The hospital workers demonstrating event-related distress, as measured by the IES-R, showed significantly higher rates of general distress, as evaluated by the GHQ-12, compared to those without event-related distress. Compared to the low-risk workers, the high-risk workers were more likely to report general distress and event-related distress. Those having actual direct contact with at least one COVID-19 patient showed significantly higher rates of general distress and event-related distress compared to those without direct contacts. Those showing the confidence in the standard precaution, were less likely to report general distress and event-related distress compared to those without confidence. Among the age groups, employees in their 40s were most likely to have both general and event-related distress.

Regarding the occupations, compared to the medical doctors, other occupations reported general and event-related distress. Especially office workers, clerks, and others showed much higher rates of event-related distress. Those living with the elderly were more likely to report event-related distress. Gender was not associated with event-related distress. However, females, as well as those with preexisting disease, were more likely to report general distress.

### Factor analysis

In terms of the stress-related 26 questions, the factor analysis revealed that 20 items loaded on six factors with factor loadings of ≥0.40 (Table 3). The label of the six factors was as follows; ‘Anxiety about infection,’ ‘Feeling of being isolated and discriminated,’ ‘Exhaustion,’ ‘Feeling of being protected,’ ‘Workload,’ and ‘Increase of exposure to TV and internet media.’

**Table 3:** Factor analysis of the 26 stress-related questions.

Questions		F1	F2	F3	F4	F5	F6
**Factor 1: Anxiety about infection** (Cronbach’s α = 0.83)							
**Q1**	Anxiety about being infected	**0.913**	-0.12	-0.006	0.013	0.023	-0.095
**Q2**	Anxiety about infecting family	**0.879**	-0.078	-0.04	0.00	0.001	-0.08
**Q7**	Lack of knowledge about infectivity and virulence	**0.665**	0.071	-0.04	0.043	0.001	0.01
**Q5**	Anxiety of being infected during commuting	**0.596**	0.042	-0.017	0.055	-0.085	0.017
**Q6**	Lack of knowledge about prevention and protection from infection	**0.53**	0.215	-0.058	0.022	0.007	-0.031
**Q21**	Heighten awareness of physical condition management	**0.517**	-0.186	0.035	0.153	0.002	0.202
**Q12**	Anxiety about compensation	**0.497**	0.07	0.007	-0.075	0.023	0.019
Q26	Doubt about uninformed serious information	0.351	0.161	0.074	-0.151	-0.067	0.105
Q11	Feeling of being supported by family	0.322	-0.028	0.085	0.274	0.019	0.017
Q20	Feeling of having no choice but to work due to obligation	0.288	-0.015	0.063	-0.032	0.065	0.064
**Factor 2: Feeling of being isolated and discriminated** (Cronbach’s α = 0.73)							
**Q14**	Feeling of being isolated	0.025	**0.802**	-0.02	0.025	-0.013	-0.059
**Q15**	Elevated mood	-0.078	**0.599**	0.008	0.265	-0.008	-0.007
**Q8**	Feeling of being avoided by others	0.057	**0.556**	-0.153	-0.019	0.071	0.025
**Q16**	Insomnia	-0.018	**0.518**	0.316	0.075	-0.072	-0.02
**Q13**	Hesitation to work	0.169	**0.418**	0.092	-0.2	-0.015	0.00
Q25	Unfairness	0.123	0.391	0.068	-0.213	0.104	0.072
Q24	Greater amount of alcohol drinking	-0.098	0.38	-0.036	0.019	0.03	0.121
**Factor 3: Exhaustion**(Cronbach’s α = 0.90)							
**Q17**	Physical exhaustion	-0.034	-0.041	**0.948**	0.015	0.039	-0.019
**Q18**	Mental exhaustion	0.026	0.045	**0.867**	0.011	-0.008	-0.012
**Factor 4: Feeling of being protected** (Cronbach’s α = 0.72)							
**Q10**	Feeling of being protected by hospital	0.024	0.008	0.056	**0.821**	0.003	-0.004
**Q9**	Feeling of being protected by national and local governments	-0.023	0.228	-0.117	**0.692**	-0.006	-0.029
Q19	Motivation to work	0.1	-0.089	0.084	0.399	0.05	0.093
**Factor 5: Workload** (Cronbach α = 0.85)							
**Q4**	Burden of change of quality of work	0.009	0.05	-0.027	0.015	**0.854**	-0.005
**Q3**	Burden of increase quantity of work	-0.018	0.007	0.056	0.028	**0.842**	-0.011
**Factor 6: Increase of exposure to TV and internet media** (Cronbach’s α = 0.79)							
**Q22**	Increase of TV exposure	-0.011	0.036	-0.041	0.036	-0.001	**0.812**
**Q23**	Increase of internet and SNS exposure	0.028	0.047	0.005	0.001	-0.016	**0.774**
	Eigenvalue	5.218	4.327	4.268	1.837	3.331	2.471
	Variance Explained (%)						47.388
	Between factor correlation						
	F2	0.458					
	F3	0.507	0.606				
	F4	-0.196	-0.177	-0.207			
	F5	0.438	0.472	0.51	-0.125		
	F6	0.435	0.26	0.262	0.057	0.178	

Abbreviation: F, Factor.

**Bold**, factor loading **≥ 0.40.**

### Logistic regression analyses

The multivariable logistic regression analyses revealed some risk factors for the general and/or event-related distress among high- or low-risk workers as follows (Table 4). The scores of the GHQ-12 were associated with event-related distress of both the high- and low-risk workers.

**Table 4:** Factors associated with general distress and event-related distress identified by multivariable logistic regression analysis.

Characteristics and Mental health outcome factors	High-risk workers (N = 528) ^a^				Low-risk workers (N = 2108) ^b^				
	β	SE	Adjusted OR ^c^ (95% CI)	P	β	SE	Adjusted OR ^c^ (95% CI)		P
**General Distress** ^**d**^									
Gender, female vs. male	0.03	0.30	1.03 (0.58–1.84)	.92	0.52	0.17	1.68 (1.21–2.34)		< .001**
Age group(years), vs. 20–29				.11					< .001**
30–39	0.75	0.33	2.12 (1.12–4.02)	.02*	0.58	0.17	1.79 (1.27–2.52)		< .001**
40–49	0.66	0.34	1.94 (1.00–3.76)	.05	0.59	0.18	1.81 (1.27–2.56)		< .001**
50-	0.72	0.42	2.05 (0.90–4.67)	.09	0.77	0.19	2.17 (1.49–3.15)		< .001**
Occupation, vs. Medical Doctor				.59					.75
Nurse	0.11	0.38	1.11 (0.53–2.35)	.78	-0.05	0.19	0.95 (0.66–1.38)		.80
Other medical professionals ^**f**^	0.23	0.38	1.25 (0.60–2.64)	.55	0.15	0.22	1.17 (0.76–1.79)		.48
Office workers and clinical clerks	0.75	0.50	2.13 (0.80–5.63)	.13	0.02	0.20	1.03 (0.70–1.50)		.90
Others ^g^	0.20	0.58	1.22 (0.39–3.79)	.73	-0.18	0.27	0.84 (0.50–1.40)		.50
Not living with partner	0.53	0.26	1.70 (1.03–2.81)	.04*	0.08	0.13	1.09 (0.85–1.39)		.51
Not confident in standard precaution	0.57	0.24	1.76 (1.10–2.81)	.02*	0.10	0.11	1.10 (0.88–1.38)		.40
Factor 1: Anxiety about infection	-0.01	0.04	0.99 (0.92–1.06)	.73	0.06	0.02	1.06 (1.02–1.10)		< .001**
Factor 2: Feeling of being isolated	0.24	0.05	1.27 (1.14–1.40)	< .001**	0.14	0.03	1.15 (1.09–1.21)		< .001**
Factor 3: Exhaustion	0.51	0.09	1.66 (1.39–1.99)	< .001**	0.66	0.05	1.92 (1.76–2.11)		< .001**
Factor 4: Feeling of being protected	-0.23	0.10	0.80 (0.65–0.97)	.03*	-0.10	0.05	0.91 (0.82–1.00)		.05
Factor 5: Workload	0.06	0.09	1.07 (0.89–1.27)	.48	-0.07	0.05	0.93 (0.86–1.02)		.13
Factor 6: Increase of TV or internet	0.13	0.08	1.14 (0.97–1.34)	.11	0.06	0.04	1.06 (0.98–1.14)		.18
	β	SE	Adjusted OR^e^ (95% CI)	P	β	SE	Adjusted OR ^e^ (95% CI)		P
**Event-related Distress** ^**f**^									
Gender, female vs. male	-0.87	0.54	0.42 (0.15–1.21)	.11	-0.94	0.32	0.39 (0.21–0.73)		.003*
Age group(years), vs. 20–29				.11					.47
30–39	0.51	0.64	1.66 (0.47–5.83)	.43	-0.21	0.38	0.82 (0.39–1.71)		.59
40–49	1.46	0.65	4.29 (1.19–15.4)	.03*	0.03	0.36	1.03 (0.51–2.07)		.93
50-	0.97	0.82	2.63 (0.53–13.0)	.24	0.31	0.38	1.37 (0.65–2.85)		.41
Occupation, vs. Medical Doctor				.88					< .001**
Nurse	0.40	0.78	1.49 (0.32–6.90)	.61	1.02	0.43	2.76 (1.19–6.40)		.02
Other medical professionals^g^	0.16	0.83	1.17 (0.23–5.99)	.85	0.65	0.51	1.91 (0.70–5.21)		.21
Office workers and clinical clerks	0.11	0.92	1.11 (0.18–6.80)	.91	1.49	0.42	4.42 (1.94–10.1)		< .001**
Others^h^	0.88	0.97	2.42 (0.36–16.0)	.36	2.20	0.51	9.00 (3.33–24.3)		< .001**
Not living with partner	1.32	0.48	3.74 (1.45–9.67)	.01*	-0.27	0.24	0.76 (0.48–1.23)		.27
Not confident in standard precaution	0.51	0.41	1.66 (0.75–3.68)	.21	-0.24	0.22	0.78 (0.51–1.22)		.28
Factor 1: Anxiety about infection	0.03	0.07	1.03 (0.91–1.18)	.62	0.12	0.04	1.13 (1.06–1.21)		< .001**
Factor 2: Feeling of being isolated	0.35	0.10	1.42 (1.17–1.73)	< .001**	0.22	0.05	1.25 (1.13–1.38)		< .001**
Factor 3: Exhaustion	0.28	0.19	1.32 (0.91–1.91)	.14	0.22	0.09	1.25 (1.04–1.49)		.02*
Factor 4: Feeling of being protected	0.05	0.19	1.05 (0.73–1.52)	.78	0.03	0.09	1.03 (0.86–1.23)		.73
Factor 5: Workload	0.07	0.17	1.08 (0.77–1.51)	.68	0.11	0.08	1.11 (0.96–1.29)		.17
Factor 6: Increase of TV or internet	0.19	0.14	1.21 (0.93–1.57)	.16	0.16	0.08	1.17 (1.01–1.35)		.04*
The scores of the GHQ-12	0.28	0.08	1.33(1.15–1.54)	< .001**	0.21	0.04	1.23(1.14–1.32)		< .001**

Abbreviations: GHQ-12, the 12-item General Health Questionnaire; IES-R, the impact of Event Scale-Revised.

^a^ Data were missing for 8 participants (1.5% of high-risk workers).

^b^ Data were missing for 53 participants (2.5% of low-risk workers).

^c^ Adjusted for gender, age group, occupation, direct exposure to COVID-19 patients, preexisting disease, living with partner, living with elderly, confident in standard precaution, and the factors1-6.

^d^ General distress evaluated by the GHQ-12 (≥4).

^e^ Adjusted for the scores of the GHQ-12, gender, age group, occupation, direct exposure to COVID-19 patients, preexisting disease, living with partner, living with elderly, confident in standard precaution, and the factors1-6.

^f^ Event-related distress evaluated by the IES-R (≥25).

^g^ Other medical professionals include clinical laboratory technicians, radiological technologists, medical engineers, pharmacists, dieticians, social workers, physical therapists, occupational therapists, and speech therapists.

^h^ Others include nursing assistants, janitors, food service, and laundry staff.

The ‘Feeling of being isolated and discriminated’ was associated with the general and event-related distress of both the high- and low-risk workers. The ‘Exhaustion’ was also related to both the general and event-related distress of the low-risk workers and the general distress of the high-risk workers. In terms of the ‘Anxiety about infection,’ this factor showed no association with the general or event-related distress of the high-risk workers. Nevertheless, it demonstrated significant relationships with both types of distress among low-risk workers.

For the high-risk workers, 30s was associated with general distress, while 40s was related to the event-related distress. ‘Not living with partner’ was a risk factor for general and event-related distress. ‘Not confident in standard precaution’ was a risk factor for general distress. On the contrary, ‘Feeling of being protected’ was negatively associated with general distress.

Regarding the low-risk workers, females and over 30 years old were risk factors for general distress. Male, office workers, clinical clerks, others, and ‘Increase of exposure to TV and internet media’ were risk factors for event-related distress.

## Discussion

This cross-sectional study enrolled 2697 respondents and revealed a high prevalence of general psychological distress among hospital workers at the beginning of the COVID-19 pandemic in Japan. We found that the ‘Feeling of being isolated and discriminated’ was an independent risk factor for worse mental health outcomes among the hospital workers, regardless of their working risks. Our study further indicated that living with partners lowered the risk of general and the event-related distress for high-risk workers. In addition, our findings indicated that increased exposure to TV and Internet media was associated with the event-related distress among the low-risk workers.

Since our study was conducted at the beginning of the pandemic, only 20.0% of all the participants were directly engaged in clinical activities with COVID-19 patients, while most employees (80.0%) were the low-risk workers. The IES-R, which measured ‘event-related distress of the COVID-19 outbreak on the Diamond Princess,’ showed that only 7.0% of the respondents experienced a high level of event-related distress; however, 35.0% of the hospital workers experienced general psychological distress, as assessed by the GHQ-12, at the threshold of 3/4 for Japanese [19, 22, 23] (45.8% of respondents showed general distress when a threshold of 2/3 was used in the GHQ-12) [18]. A Canadian study demonstrated that 29.0% of the hospital workers experienced severe general psychological distress (evaluated by the GHQ-12 with the threshold of 2/3) during the outbreak of SARS [27]. Another study reported that psychiatric morbidity on the IES and the GHQ-28 were 17.7% and 18.8% among the hospital workers, respectively at six months after the peak of the SARS pandemic in Singapore [28]. It seems difficult to make direct comparisons between our findings of the IES and the GHQ and those of previous studies due to the different evaluation times. However, our results revealed the discrepancy between scores of the IES-R and GHQ-12 among the hospital workers at the beginning of the COVID-19 pandemic. In our hospital workers, the severity of general distress, i.e., the scores of the GHQ-12, could reflect pre-existing distress due to hard hospital works and newly added distress due to the COVID-19 outbreak. This discrepancy suggests that the hospital workers had already shown general psychological distress before they had actual contact with infected patients, which might be based on their daily hard work, anxiety and fear of COVID-19 infection itself, and/or fear of something unknown about COVID-19 [29]. Since scores of the GHQ-12 and the IES-R were related, the general distress could be the risk factor for the event-related distress.

This study revealed that ‘Feeling of being isolated and discriminated’ was associated with general psychological distress and event-related distress for both the high- and low-risk workers. A previous study of Canadian hospital workers has reported that 28% of the hospital workers felt “being treated differently because of working in hospital” during the outbreak of SARS [27]. A study from Singapore also reported that 31% of the healthcare workers perceived that “people avoid my family members because of my job” during the SARS outbreak [30]. Other studies on SARS have also shown that “Social isolation and avoidance” [24], “Perceived stigma” [5], and “Being discriminated as high-risk spreader” [31] could be sources of stress. Similar results have been demonstrated in studies of other disasters where ‘Discrimination’ was a risk factor for worsened post-disaster mental health among workers at Fukushima nuclear power plants [25] and Vietnam War veterans [32]. The previous and current results suggest that ‘Feeling of being isolated and discriminated’ could adversely affect the mental health of workers at any point during a disaster (e.g., at the beginning of, during, and after the disaster); thus, it is necessary to establish an educational program to prevent discrimination against hospital staffs working under the threats, such as COVID-19 infection. Then, the program should be disseminated to all the citizens, including hospital workers, from the early stage of the pandemic.

Contrary to previous studies [24, 27], our study indicated that ‘living with partner’ was significantly associated with lower scores on the IES-R and GHQ-12, especially among the high-risk workers. We think that the availability of partners or families might have buffering effects on the distress of high-risk workers despite their worries about passing infection at the beginning of the pandemic.

Furthermore, we found that office workers, clinical clerks, and others (e.g., nursing assistants, janitors, food service, and laundry staff) scored high on IES-R, especially those in the low-risk group. Previous studies have been reported repeatedly that nurses experience the highest mental distress among all occupations in hospitals during the outbreak of SARS and H1N1 influenza [5, 7, 33]. However, one study revealed higher levels of anxiety among the support staff as opposed to the medical doctors and the nurses [34], and this research was conducted relatively early during the outbreak of SARS, similar to our current study. We assume that office workers, clinical clerks, and others might have had insufficient protective equipment and information about COVID-19 infection because of their low-risk work environment. Thus, they continued to be scared of the mysterious COVID-19 infection, resulting in high IES-R scores at the beginning of the outbreak.

The ‘Increase of exposure to TV and internet media’ was also associated with increased IES-R scores among the low-risk workers. In general, watching more TV has been generally associated with more severe mental distress, as watching TV before going to bed might disrupt sleep quality and trigger mental dysfunction [35, 36]. In the case of COVID-19, TV and Internet media featured the Diamond Princess and the increasing number of infected people daily; thus, all the hospital workers might have felt increased anxiety and fear of admitting patients with COVID-19 in addition to the infection itself at the beginning of the outbreak [37]. The high-risk workers encountered real patients while the low-risk workers did not; therefore, they had limited access to actual and concrete information, which could have increased their anxiety. Therefore, watching TV programs about the outbreak of COVID-19 in the Diamond Princess could be a risk factor for developing trauma-like responses among the low-risk workers.

This study has several limitations worth noting. First, the response rate in our research was 66.9%, which is relatively good for paper-based, self-administered, anonymous questionnaires. However, response bias may still have been present if the non-respondents were either too stressed to respond or not stressed at all and, therefore, not interested in this survey. Second, this study was unable to distinguish pre-existing mental health symptoms from new symptoms. We are designing a further study that will be conducted after the pandemic in the same hospitals to evaluate longitudinal changes in mental health status in our hospital workers. Third, the validity of the questions assessing COVID-19 related stress has not been adequately established, since they are partly original questions, which is another limitation. Fourth, this study was carried out from March 23 to April 6, and the COVID-19 situation in Japan changed to worse during the subsequent 15 days. Hence, the mental situation might have changed from the beginning to the end of this period. However, we believe that we could evaluate the mental health status of our hospital workers just at the beginning of the pandemic, and we think this report is important in this regard.

## Conclusions

In this survey study, workers in the hospitals who responded to the patients with COVID-19 reported high rates of general psychological distress and a certain degree of the event-related traumatic distress at the beginning of the pandemic in Japan. The daily hard work may have contributed to their general distress, which could become the risk factor of event-related distress. We found that isolation and discrimination were independent risk factors for worse mental health outcomes among the hospital workers, regardless of their working risks. Thus, it is necessary to prevent discrimination against hospital staffs from the early stage of the pandemic. Special mental health support for not only high-risk workers but also low-risk workers, especially with increased exposure to TV and Internet media, needed to be promptly implemented from the beginning of the outbreak.
